# A novel mutation in the sterol 27-hydroxylase gene of a woman with autosomal recessive cerebrotendinous xanthomatosis

**DOI:** 10.1186/1750-1172-5-27

**Published:** 2010-10-06

**Authors:** Hauke Schneider, Alexandra Lingesleben, Hans-Peter Vogel, Rita Garuti, Sebastiano Calandra

**Affiliations:** 1 University of Technology Dresden, University Clinic, Department of Neurology, Fetscherstr. 74, 01307 Dresden, Germany; 2 Vivantes August-Viktoria-Klinikum, Department of Psychiatry and Psychotherapy, Rubensstraße 125, 12157 Berlin, Germany; 3 Cecilie-Vogt-Clinic of Neurology, Helios-Klinikum Berlin-Buch, Schwanebecker Chaussee 50, 13125 Berlin, Germany; 4 Promega Corporation, 2800 Woods Hollow Road, Madison, WI 53711, USA; 5 Department of Biomedical Sciences, University of Modena, Via del Pozzo 71, 41124 Modena, Italy

## Abstract

**Article abstract:**

Mutations of the gene encoding the mitochondrial enzyme sterol 27-hydroxylase (*CYP27A1 *gene) cause defects in the cholesterol pathway to bile acids that lead to the storage of cholestanol and cholesterol in tendons, lenses and the central nervous system. This disorder is the cause of a clinical syndrome known as cerebrotendinous xanthomatosis (CTX). Since 1991 several mutations of the *CYP27A1 *gene have been reported. We diagnosed the clinical features of CTX in a caucasian woman. Serum levels of cholestanol and 7α-hydroxycholesterol were elevated and the concentration of 27-hydroxycholesterol was reduced. Bile alcohols in the urine and faeces were increased. The analysis of the *CYP27A1 *gene showed that the patient was a compound heterozygote carrying two mutations both located in exon 8. One mutation is a novel four nucleotide deletion (c.1330-1333delTTCC) that results in a frameshift and the occurrence of a premature stop codon leading to the formation of a truncated protein of 448 amino acids. The other mutation, previously reported, is a C - > T transition (c. c.1381C > T) that converts the glutamine codon at position 461 into a termination codon (p.Q461X). These truncated proteins are expected to have no biological function being devoid of the cysteine residue at position 476 of the normal enzyme that is crucial for heme binding and enzyme activity.

## Introduction

Cerebrotendinous xanthomatosis (CTX) (OMIM 213700; ORPHA 909) [1,2] is an autosomal recessive sterol storage disease caused by mutations of the sterol 27-hydroxylase-gene (CYP 27). The *CYP27A1 *gene is located on chromosome 2q33-qter and consists of 9 exons. Sterol 27-hydroxylase is a mitochondrial cytochrome P 450 enzyme that catalyzes the initial steps in the oxidation of side chain of sterol intermediates in the pathway leading to the formation of bile acids in the liver [3-5]. In CTX patients the ability to convert cholesterol to bile acids is impaired and the incomplete oxidation of the cholesterol side chain leads to the accumulation of cholesterol and abnormal tetra-and penta-hydroxylated bile alcohols in tendons, lenses, the central nervous system and lungs. Children with CTX will often have diarrhea of unknown cause and develop juvenile cataracts and tendon xanthomas [6]. Premature atherosclerosis has also been observed. The characteristic neurological features often leading to diagnosis in the second or third decade of life are pyramidal tract signs, cerebellar syndrome, peripheral neuropathy, epileptic seizures, speech- and swallowing-disorders.

Currently, about 50 mutations of the *CYP27A1 *gene are known [7-11], mainly affecting the adrenodoxin-binding and heme binding-sites of the sterol 27-hydroxylase. Here we describe clinical findings, biochemical parameters and a novel mutation of the *CYP27A1 *gene in a woman with CTX.

## Case presentation

The patient, a 35 year-old german female, complained of a progressive disturbance of gait over the last 3 years. She had a history of recurrent diarrhea and bronchitis, growth retardation, a mild retardation of psychomotor development in infancy and bilateral juvenile cataracts. She attended a school for mentally and physically handicapped children. At age 24 a normal pregnancy and delivered an apparently healthy child. Another child died four days after birth. There was no information about the proband's father so that consanguinity or clinical features consistent with CTX in father's family cannot be excluded. The patient's mother and daughter as well as other known family members have no clinical symptoms or signs of CTX.

Detailed physical examination revealed significant findings of bilateral swelling of the Achilles tendons and high arched feet. The patient had a tetraspastic syndrome with exaggerated tendon reflexes including the masseter reflex, positive bilateral extensor plantar responses and an unsteadiness of gait with difficulties in walking in tandem. There was no Romberg sign. The vibration sense was mildly impaired in the lower limbs. Psychological testing revealed a slowing in psychomotor activity with decrease of concentration and difficulties in visual and abstract learning.

Routine laboratory tests were within normal limits. Serum cholesterol was 143 mg/l and serum cholestanol was 25.8 mg/l; cholestanol:cholesterol ratio was 1: 55 and increased to six times of normal limit; bile acid precursor 7α-hydroxycholesterol was 5068 ng/ml (normal range 70-100 ng/ml) and 27-hydroxycholesterol 13 ng/ml (normal range 70-100 ng/ml). Acid steroids and bile alcohols in urine and faeces were also elevated. CSF examination showed increased protein (611 mg/l) with normal cell count, glucose and lactate levels.

The EEG revealed a posterior 4-5/s rhythm and additionally frontal intermittent rhythmic delta activity (FIRDA). Somatosensory evoked potentials (tibialis nerve) revealed borderline values for response at N22 (lumbar spinal cord) and a delayed response at P40 (cortex) bilaterally of 50.6 ms and 51.1 ms indicating a central demyelination. Nerve conduction studies showed moderate reduction of motor conduction velocities (tibial and peroneal nerve, 33-38 m/sec) suggesting mild demyelination in the peripheral nervous system. Central motor conduction times to tibialis anterior were prolonged (23.1 ms and 24.5 ms) indicating demyelination of central motor pathways. MRI of head und cervical spine was normal and MRI of Achilles tendons showed confluent lipid inclusions.

Treatment with chenodeoxycholic acid was initiated (750 mg/day). Three years later physical examination revealed no progression of clinical manifestations. Serum concentrations of cholesterol and cholestanol were within normal limits. Nerve conduction studies showed normal motor conduction velocities (tibial and peroneal nerves) and central motor conduction times were slightly prolonged to tibialis anterior (21,4 ms and 22,9 ms). EEG was improved with alpha rhythm and without FIRDA. MRI of Achilles tendons showed unchanged lipid inclusion.

## Analysis of *CYP27A1 *Gene

Genomic DNA was extracted from peripheral blood leukocytes by a standard procedure [12,13]. The promoter region and the exons of CYP27 gene were amplified by PCR from genomic DNA using the primers reported previously by Leitersdorf et al. [7]. PCR products were sequenced directly (Thermosequenase kit, USB Amersham, UK.). For the sequence of the exon 6 - exon 9 region we used the following oligonucleotides: 5' GAG ATC CAG GAG GCC TTG CAC GA 3' (forward primer in exon 6 corresponding to nucleotides 1066-1089 of the cDNA) (6S); 5' GAT TGG GCA GCA TGA ATG CCA CTC 3' (reverse primer complementary to nucleotides 75-52 of intron 7) (7B); 5' CCC AGT TTG TGT TCT GCC AC 3' (forward primer in exon 8 corresponding to nucleotides 1265 - 1285 of the cDNA) (8S); 5' GGA GTA GCT GCA TCT CCA GCT CT 3' (reverse primer in exon 8 complementary to nucleotides 1468 - 1447 of the cDNA) (8AS); 5' CCC AGC AAG GCG GAG ACT CA 3' (reverse primer in the 3' untranslated region complementary to nucleotides 1618 - 1600 of the cDNA) (9B).

To confirm the presence of the mutations in exon 8 of *CYP27A1 *gene found by sequencing (see below) the exon 6 - exon 9 region was amplified by PCR and the fragment digested with the restriction enzymes Hind III or BamHI. The restriction fragments were separated by 1% agarose gel electrophoresis.

## Mutations in *CYP27A1 *Gene

**Figure 1:**
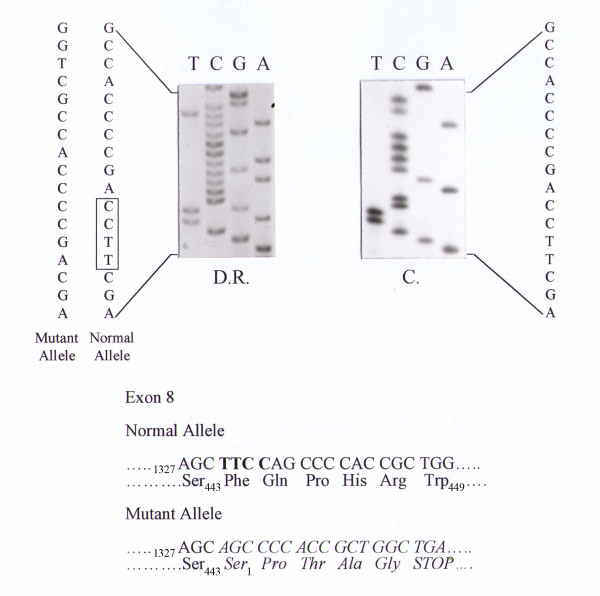
**Partial nucleotide sequence of exon 8 in proband D.R. and in a control subject (C.)**. Patient is heterozygous for a 4 nucleotides deletion (boxed) (c.1330-1333delTTCC). This deletion causes a shift in the reading frame resulting in a string of five novel amino acids (in italics) and the occurrence of a premature stop codon.

**Figure 2:**
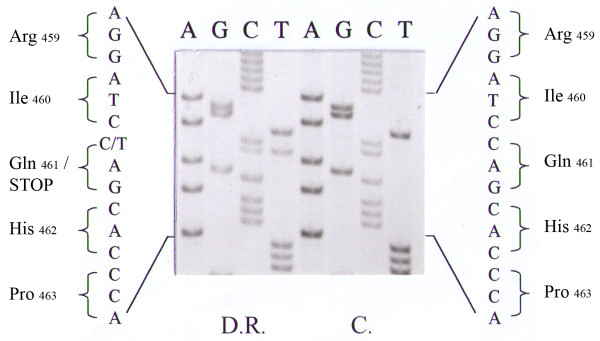
**Partial nucleotide sequence of exon 8 in patient and in a control subject (C.)**. Patient is heterozygous for C- > T transition at CYP cDNA position c.1381 (p.Q461X).

Figures 1 and 2 show that the patient was a compound heterozygote carrying two mutations in exon 8: i) a 4 nucleotide deletion (TTCC) (c.1330-1333 del TTCC) and ii) a C- > T transition (c.1381C > T). The four nucleotide deletion causes a shift in the reading frame downstream of the codon for serine (p.S443) leading to a sequence of five novel amino acids preceding a premature stop codon (p.S443 > Fs > X449). The presence of the four nucleotide deletion was confirmed by restriction fragment analysis, as this deletion eliminates a HindIII restriction site. The digestion of exon 6 - exon 9 region generated two fragments of 631 bp and 445 bp respectively in control DNA and a single fragment of 1076 bp in patient's DNA.

The second mutation is a non-sense mutation which converts the codon (CAG) for glutamine (p.Q461) into a stop codon. This mutation was previously reported as Q428X in the mature protein [10] or Q461X in the pre-protein (that includes the 33 amino acid of the mitochondrial signal peptide) [11]. The presence of the mutation was confirmed by restriction fragment analysis, due to the elimination of BamHI restriction site. The digestion of exon 6 - exon 9 region generated two fragments of 681 bp and 395 bp respectively in control DNA and a single fragment of 1076 in patient's DNA.

Patient's daughter was heterozygote for the 4 nucleotide deletion (data not shown).

## Conclusion

Two decades after the first report describing defects in sterol 27-hydroxylase as underlying cause of the autosomal recessive disease cerebrotendinous xanthomatosis (CTX), several mutations in the *CYP27A1 *gene have been described [11].

The sequence analysis of *CYP27A1 *gene in our CTX patient revealed two mutations, leading to the formation of truncated proteins. The first mutation is a deletion of four nucleotides in exon 8 (c.1330-1333del TTCC) generating a frameshift with a premature termination codon and resulting in a truncated protein of 448 amino acids. To our knowledge this is the first 4 nt deletion reported in CYP27 gene in a CTX patient. In other genetic diseases this deletion occurs in a context of DNA sequence characterized by several direct or inverted short nucleotide repeats [14]. The presence of these repeats is thought to be cause for slipped mispairing at the DNA replication [15] that, in turn, may produce minute deletions or insertions.

The second mutation is a C - > T transition at cDNA position c.1381 (exon 8) that converts codon CAG (p.Q461X) into TAG (stop) leading to a truncated protein of 460 amino acids.

The truncated proteins encoded by the mutant alleles found in our patient are devoid of the cysteine at position p.476 of the normal enzyme, which has been shown to be crucial for heme binding and enzyme activity [16]. Therefore, although we have not done *in vitro *expression studies with mutant cDNAs, it is most likely that lack of a functional heme binding domain in the truncated proteins results in a decreased activity of the sterol 27-hydroxylase and the CTX phenotype of our patient.

DNA analysis of the patient's daughter revealed that she is heterozygous for the 4 nucleotide deletion. Although she is expected to have a 50% reduction of sterol 27-hydroxylase activity in liver and other tissues, she is free of symptoms as it is usually the case in carriers of a single mutation of *CYP27A1 *gene. It is supposed that only a reduction of enzyme activity greater than 90% results in an increased production of metabolites like cholestanol or bile alcohols, partially responsible for the clinical phenotype in CTX subjects [17].

We know that both of the patient's parents are of the same caucasian background and her mother is free of clinical signs associated with CTX. Further diagnostic studies and genetic analyses of the patient's family were declined and therefore it remains unclear whether our patient received these mutations from her parents or carries "de novo" mutations.

Several compound heterozygous patients have been described [7,18-23]. As pointed out in a retrospective study evaluation of these patients and the comparison with individuals homozygous for CTX mutations revealed no clear genotype-phenotype relations [8]. This is consistent with earlier findings suggesting a strong influence of environmental and other genetic factors on the development of individual CTX phenotypes [21].

Knockout mice deficient in the sterol 27-hydroxylase (cyp27^-/-^) have been generated by gene targeting [24]. The disruption of the sterol 27-hydroxylase gene in mice did not lead to neurological, metabolic or vascular alterations similar to those observed in CTX patients most likely due to species differences in the bile acid and cholesterol metabolism. The CYP27 -/- mice therefore are not a suitable model for investigating the pathophysiology of sterol accumulation in tissues in CTX patients.

## Abbreviations

CTX: cerebrotendinous xanthomatosis; CSF: cerebrospinal fluid; EEG: electroencephalogram; FIRDA: frontal intermittent rhythmic delta activity; MRI: magnetic resonance imaging

## Consent

Written informed consent was obtained from the patient for publication of this case report. A copy of the written consent is available for review by the Editor-in-Chief of this journal.

## Competing interests

The authors declare that they have no competing interests.

## Authors' contributions

HS, AL and HPV participated in clinical evaluation and drafting the manuscript. RG and SC carried out genetic studies and helped to draft the manuscript. All authors read and approved the final manuscript.
